# Quinoa (*Chenopodium quinoa* Willd.) Saponins and Their Pharmaceutical Potential: A Review

**DOI:** 10.3390/ijms27156679

**Published:** 2026-07-27

**Authors:** Stella Karydogianni, Ioannis Roussis, Myrto Chatzitriantafyllou, Stavroula Kallergi, Panteleimon Stavropoulos, Antonios Mavroeidis, Dimitrios Bilalis, Ioanna Kakabouki

**Affiliations:** Laboratory of Agronomy, Department of Crop Science, Agricultural University of Athens, 11855 Athens, Greece; roussis@aua.gr (I.R.); stud120006@aua.gr (M.C.); kallergi@aua.gr (S.K.); stavropoulosp@aua.gr (P.S.); antoniosmauroeidis@gmail.com (A.M.); bilalis@aua.gr (D.B.); i.kakabouki@aua.gr (I.K.)

**Keywords:** anticancer activity, *Chenopodium quinoa*, Chenopodiaceae, saponins

## Abstract

This review provides an updated and comprehensive assessment of the current literature on quinoa (*Chenopodium quinoa* Willd.) saponins, with particular emphasis on pharmacological effects. Quinoa (*Chenopodium quinoa* Willd.) is an annual plant native to South America. Quinoa seeds are characterized by high nutritional value and are rich in proteins, lipids, carbohydrates, minerals, and saponins. Saponins are found in quinoa seeds and impart a bitter taste, which is why they are typically removed before seed consumption. Saponins have been characterized as antinutritional agents, but in recent years they have been investigated for their pharmaceutical applications. Protocols have been developed for the extraction of saponins from seeds, such as conventional solid–liquid extraction (maceration), ultrasound-assisted extraction, microwave-assisted extraction, enzyme-assisted extraction, and pressurized liquid extraction. Ultrasound-assisted extraction and microwave-assisted extraction are considered the most suitable methods for quinoa saponins. Quinoa saponins have shown promise as vaccine adjuvants. Furthermore, they have demonstrated direct anticancer activity, inducing apoptosis and inhibiting the proliferation of breast and colon cancer cells. In general, saponins can induce hemolysis at high concentrations through membrane disruption, primarily via lipid solubilization or pore formation. However, hemolytic activity varies significantly among different saponins and depends on their chemical structure and concentration. In vivo, they have not recorded adverse side effects at doses below 50 mg/kg body weight per day. More than 40 triterpenoid saponins, mainly derived from oleanolic acid, ederagenin, phytolaccagenic acid, and sergianic acid, have been identified in quinoa. Overall, although quinoa saponins have traditionally been considered antinutritional compounds, accumulating evidence indicates that they possess promising pharmacological properties, particularly as anticancer agents.

## 1. Introduction

**Figure 1:**
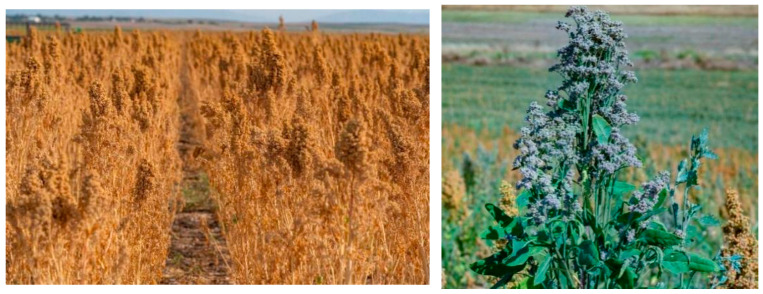
Quinoa (*Chenopodium quinoa* Willd) plants at the maturation stage and the vegetative stage from experimental crops in the Thessaly region. (Source: Laboratory of Agronomy, Agricultural University of Athens).

**Figure 2:**
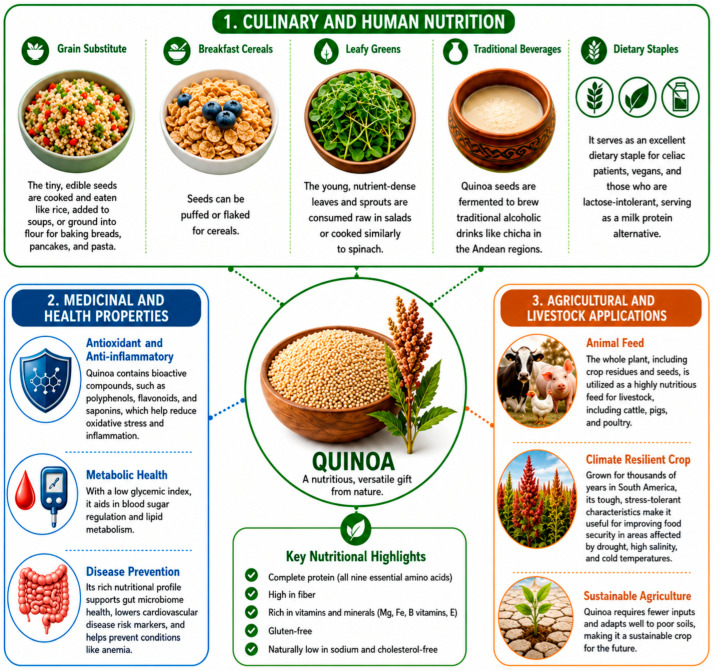
Infographic depicting the uses of quinoa. (Note: Figure generated using ChatGPT (OpenAI, GPT-5.5) and adapted by the authors).

Quinoa (*Chenopodium quinoa* Willd.) is a native annual plant of the Andean region of South America (Figure 1). The first records of its cultivation date back to 5000–3000 BC, where it was consumed as a sacred food in the Inca civilization [1]. It is an herbaceous, halophytic plant belonging to the Chenopodiaceae family and the Chenopodium genus [2]. Quinoa has been classified as a pseudocereal for botanical reasons but mainly due to its rare composition and the balance observed between lipids, proteins, and carbohydrates [3]. In recent decades, quinoa production has been steadily increasing. The Food and Agriculture Organization of the United Nations (FAO) declared in 2013 the International Year of Quinoa, during which its production and consumption increased [4]. It has attracted increasing global attention, not only because of its nutritional and functional properties but also because of its ability to be cultivated under adverse climatic conditions [5]. Quinoa is considered a functional food (super food), and the seed is used whole or as flour to produce bread, cakes, cookies, pasta, couscous, etc. (Figure 2) [6]. The leaves and young shoots, due to the increased levels of protein and nutrients (Ca, P, and Fe), are superior as salads to other vegetables [7]. The green stems and leaves are used as animal feed and also dried as biofuel. The saponins, from the seed coat, can be exploited for industrial and biomedical purposes [8,9]. Quinoa is widely cultivated in South America, Peru, Asia, and Africa, and in recent years, cultivated areas have also been established in Europe [10]. It is described by the Food and Agriculture Organization of the United Nations (FAO) as the “perfect and strategic food” for human consumption [11].

Consuming products derived from quinoa seeds have been associated with the risk of certain types of cancer, lower blood sugar levels, reduced body weight, and lower blood lipid levels [12,13].

Quinoa seeds have high nutritional value and are rich in proteins, lipids, carbohydrates, minerals, and saponins [14]. They also contain flavonoids and phenolic compounds, which contribute to their antioxidant capacity [15].

Micronutrients such as potassium, phosphorus, and magnesium are contained in high concentrations in quinoa, covering daily human needs [16]. Quinoa is also high in linoleic and linolenic fatty acids and contains antioxidants levels, reported to be up to five times higher than those of cereal flours [17].

Also, quinoa has received particular attention in recent years as an alternative to cereals, such as wheat, rye, and barley, which contain gluten proteins, while quinoa is classified as a pseudocereal and gluten-free [18]. Quinoa has been recognized for its ability as a functional food that may also have medicinal uses. Its seeds contain high levels of diverse secondary metabolites with important pharmacological activities [19]. It has been reported that quinoa improves metabolic health and may have a beneficial effect on conditions such as diabetes and cardiovascular diseases [20]. However, many antinutritional compounds are found in quinoa, such as saponins, phytic acid, tannins, and trypsin inhibitors. The saponins contained in quinoa have a bitter taste, which affects the palatability and digestibility of the food [21]. In many plant species, saponins are found as glycosylated secondary metabolites, including major food crops. The structural composition of saponins is a linkage of a hydrophilic carbohydrate chain to a triterpenoid aglycone [22]. They are characterized by a wide range of biological properties [23]. At high concentrations, saponins can cause cell lysis by disrupting cell membranes. This effect has been observed in erythrocytes, other animal cells, fungi, and bacteria. Quinoa saponins have been shown to have significant antifungal activity [24].

In this literature review, we critically examine and evaluate the potential medicinal properties of saponins derived from quinoa seeds. Compared to previous reviews [25,26], we provide an updated overview of quinoa saponins, including extraction methodologies. This review is based on the premise that quinoa saponins traditionally regarded as antinutritional compounds possess significant pharmaceutical potential and may represent promising options for biomedical applications.

## 2. Quinoa Seeds

**Figure 3:**
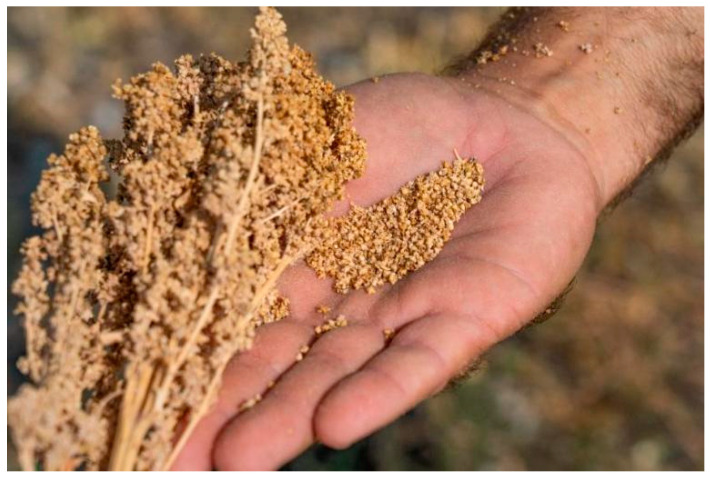
Quinoa seeds (*Chenopodium quinoa* Willd). (Source: Laboratory of Agronomy, Agricultural University of Athens).

Quinoa seeds (*Chenopodium quinoa* Willd.) usually contain saponins in the seed coat [27]. The length and diameter of quinoa seeds are on average 2.5 mm and 1.0 mm, respectively. There are varieties with different seed colors, such as yellow (Figure 3), red, brown, or black seeds [28]. Triterpene saponins have been found in all vegetative parts of the plant as well as in the seeds. However, the concentration varies depending on the variety and environmental conditions of the plant and ranges from 0.01% to 4.65% of the dry matter [29].

The protein content in quinoa seeds ranges from 14 to 16% [30]. The seeds contain a high-quality vegetable oil suitable for human consumption, where the fatty acid composition resembles the composition of soybean oil [31,32,33]. The anti-inflammatory [34] and antimicrobial effects of quinoa extracts, which have been documented in research results, are remarkable [35]. Additionally, it has been found to prevent fungal and viral diseases [36]. Quinoa has also been reported to reduce blood cholesterol levels. Furthermore, quinoa extracts have been reported to enhance the absorption of drugs from the mucosa [37].

The nutritional composition of quinoa seeds is highly valuable for human nutrition. As shown in Table 1 and Table 2, quinoa is an exceptionally balanced plant-based food, rich in both macronutrients and micronutrients. A brief overview of this composition is provided below. Given that quinoa saponins are primarily located in the seed coat, understanding the nutritional composition of quinoa seeds provides the necessary background for evaluating the presence, composition, and biological significance of these compounds. Consequently, Section 3 focuses on the chemical characterization of the saponins identified in quinoa seeds.

Carbohydrates are the predominant macronutrient in quinoa seeds. The largest percentage of carbohydrates comes from starch, which is characterized by a low glycemic index. This property contributes to a greater feeling of satiety and stabilization of blood glucose levels.

Quinoa proteins include albumins and globulins, which are particularly rich in lysine, an essential amino acid that is often limited in most cereals, making it a highly nutritious plant source.

Fiber also has high concentrations, levels comparable to those of legumes. The lipid content is considered relatively high. However, the majority consists of unsaturated fatty acids, which are associated with cardioprotective effects and better metabolic health.

Regarding minerals, quinoa stands out for its high potassium content and contributes to the regulation of blood pressure. Phosphorus and magnesium are also found at high levels, supporting the functioning of the nervous system and muscle activity and maintaining bone health. At the same time, the presence of iron and zinc gives it significant nutritional value, as these elements participate in hematopoiesis, immune function and multiple metabolic processes.

## 3. Chemical Characterization of Quinoa Saponins

**Figure 4:**
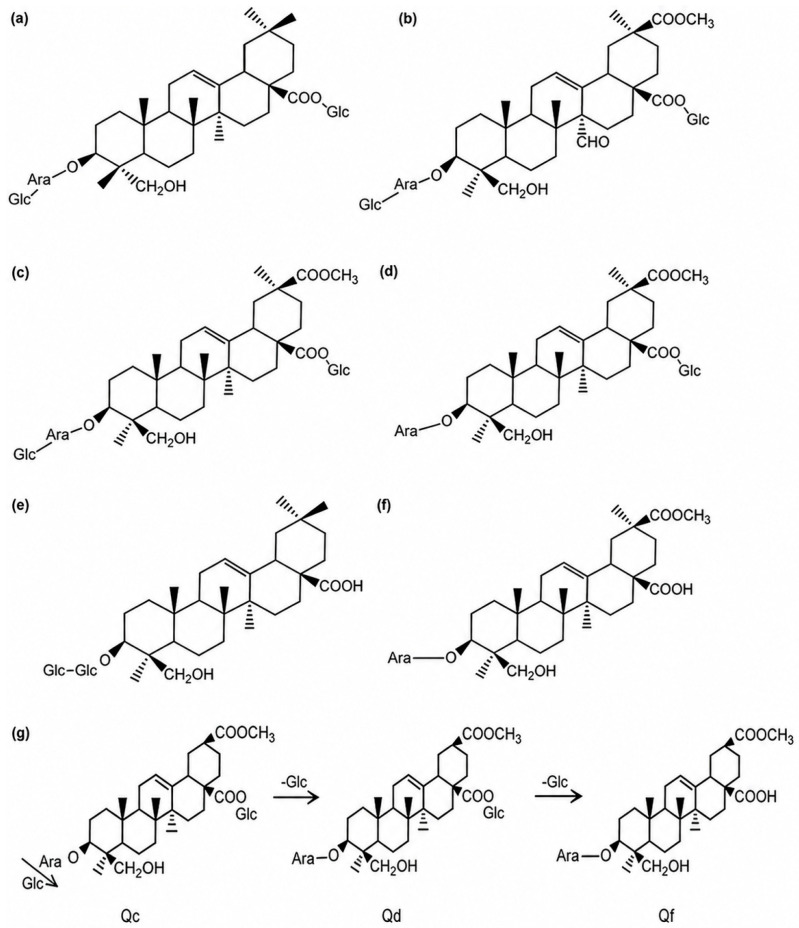
Representative chemical structures of quinoa saponins. (**a**–**f**) Major triterpenoid saponins identified in quinoa seeds, including derivatives of hederagenin and phytolaccagenic acid. (**g**) Hydrolysis process of polar quinoa saponins (from Qc to Qe and Qf) [46].

Saponins are a group of glycosides found in various vegetative parts of a wide variety of plant species. They are polar molecules with a steroid or triterpene aglycone and one or more sugar chains, linked at C-3 and C-28, which have the ability to exhibit surface-active properties [41]. Sugar chains may contain glucose, galactose, arabinose, glucuronic acid, or xylose [42,43,44]. The saponins identified in quinoa are primarily triterpenoid saponins derived from oleanane-type aglycones, including derivatives of hederagenin and phytolaccagenic acid. Representative structures of quinoa saponins are shown in Figure 4. Saponins are categorized into two groups: saponins that have a steroid aglycone and are found mainly in angiosperm monocotyledonous plant species and saponins that have a triterpene aglycone and are found in angiosperm dicotyledonous plants; the latter category includes quinoa [23]. In the outer shell of quinoa seeds, there are saponins, which are the main secondary metabolites [45].

Genotypes significantly influence the amount of saponins in quinoa. It has been found to be higher in bitter-tasting varieties compared to sweet varieties, which have low saponin content [47].

The growth stage of the crop affects the saponin content, as it is low during branching and high during flowering [48]. Salinity increases the content, while drought reduces saponin accumulation in quinoa seeds by 45% [49,50,51].

Saponins have a protective role in seeds against birds, insects, and fungi [52]. At least six different types of saponins are contained in quinoa [53], which are mainly derived from aglycones, oleanolic acid, and edragenin or phytolaccagenic acid [54]. Saponin imparts a bitter taste. The amount of saponin ranges between 0.1 and 5% [55]. Based on saponin content, quinoa varieties containing ≤0.11% saponins are classified as “sweet”, while those with higher saponin levels are considered “bitter”. It has been characterized as an antinutritional factor in quinoa despite its medicinal use. The medicinal effects of saponins include antifungal activity, where it causes structural membrane damage associated with steroid membranes.

Saponins, in addition to conferring defense mechanisms on plants, also possess a variety of biological and pharmacological properties. Such activities include hemolytic, cytotoxic, immunomodulatory, anti-inflammatory, and anticancer effects. Saponins have been used in medicine for centuries as a component of herbal medicines to combat diseases [22].

## 4. Extraction Methods of Quinoa Saponins

The traditional method of removing saponin from quinoa seeds involves washing the seeds (wet method) before cooking. However, mechanical peeling by friction (dry method) also seems to be effective [56]. Over the years, other protocols have been created, which will be analyzed below. The wet method is more expensive compared to the dry method, increasing the cost (due to drying the product). The dry method has a significant disadvantage that it does not have the efficiency to extract all the saponins, and if the efficiency is increased, it results in the loss of some nutrients [16].

Solid–liquid extractions are based on the contact of an insoluble solid material with appropriate organic and/or inorganic solvents. During this process, the extractable compounds are transferred from the solid material to the solvent, resulting in the production of a liquid extract, which, in the case of quinoa, contains the saponins of the seeds [57].

### 4.1. Conventional Solid–Liquid Extraction (Maceration)

Maceration is a simple and economical technique; the process is carried out in steel containers, with the capacity ranging from a few liters to large quantities at the industrial level, and inert materials both towards the solid material and towards the extraction solvent. Maceration is the first and oldest technique based on diffusion and osmosis. For the extraction of active ingredients from medicinal plants, it is considered the reference technique for many applications [58].

The extraction process is time-consuming, requiring days to weeks to complete. The solid material is placed in a container and completely immersed in the extraction solvent. For optimal extraction of the compounds, the container must be tightly closed and agitated.

**Figure 5:**
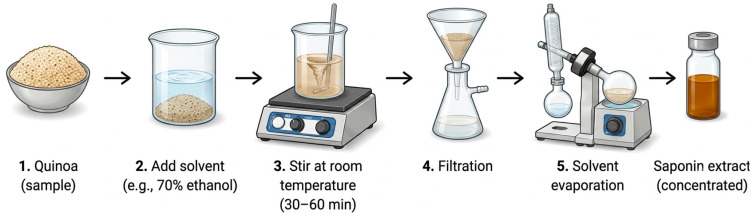
Infographic of conventional solid–liquid extraction. (Note: Figure generated using ChatGPT (OpenAI, GPT-5.5) and adapted by the authors).

Quinoa saponins are extracted by soaking the material in a solvent at room temperature with stirring for a specific time. This is followed by solid–liquid separation and solvent removal (Figure 5).

### 4.2. Ultrasound-Assisted Extraction (UAE)

**Figure 6:**
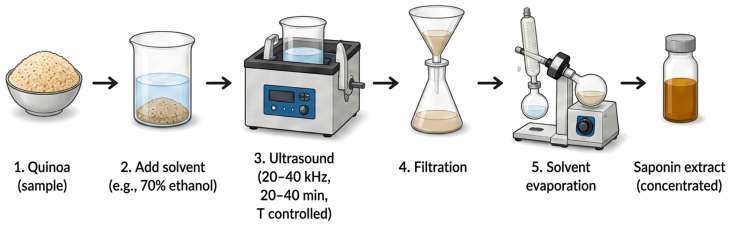
Infographic of ultrasound-assisted extraction. (Note: Figure generated using ChatGPT (OpenAI, GPT-5.5) and adapted by the authors).

Ultrasound is widely used for the extraction of polyphenols, carotenoids, volatile compounds, and polysaccharides from plant tissues. For optimal extraction and a quality final product, the following parameters must be controlled accurately: frequency, power, duty cycle, temperature, time, solvent type, and solid-to-liquid ratio (Figure 6) [59].

Ultrasonic extraction (UAE) is a process that uses ultrasonic energy and solvents to extract saponins from quinoa seeds [60]. Ultrasound is a mechanical wave that has a frequency (>20 kHz) higher than the human auditory range (20 Hz to 20 kHz). Ultrasound has the effect of increasing the swelling index of the seeds. Therefore, it helps in both the desorption and diffusion of saponins, resulting in an increased amount of extract [61].

### 4.3. Microwave-Assisted Extraction (MAE)

This method is an advanced technique for extracting active ingredients from medicinal plants, such as saponin from quinoa seeds. Using microwave energy, the solvents containing the samples are heated, thus extracting the saponins. The main advantage of this extraction method is that it heats the solvent mixture quickly, resulting in rapid extraction of the saponins [62].

**Figure 7:**
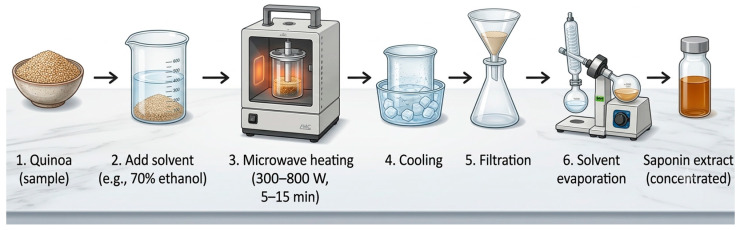
Infographic of microwave-assisted extraction. (Note: Figure generated using ChatGPT (OpenAI, GPT-5.5) and adapted by the authors).

Microwaves heat the solvent and plant matrix rapidly, increasing rupture and mass transfer, resulting in faster and more efficient extraction (Figure 7).

### 4.4. Enzyme-Assisted Extraction (EAE)

Enzymes, used in extraction, have the ability to isolate compounds of high purity. Enzyme extraction is quite expensive and therefore constitutes a significant limitation of its use. The pH and temperature of the enzyme must be checked at regular intervals because they are essential elements for ensuring optimal enzyme activity [63].

**Figure 8:**
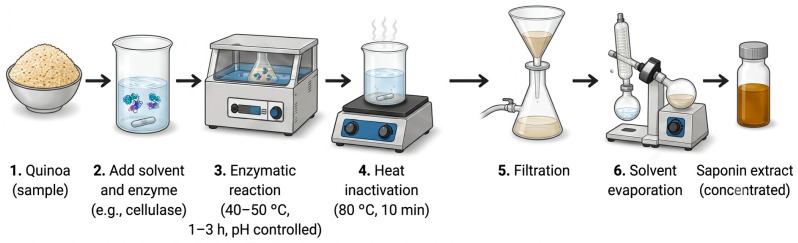
Infographic of enzyme-assisted extraction. (Note: Figure generated using ChatGPT (OpenAI, GPT-5.5) and adapted by the authors).

Enzymes hydrolyze cell wall components (e.g., cellulose, hemicellulose, and pectin), releasing bound saponins and improving extraction yield (Figure 8).

### 4.5. Pressurized Liquid Extraction (PLE)

Pressurized liquid extraction is an ecological technology for extracting nutrients from food and plant materials [64]. Pressure extraction is a rapid extraction method that uses high pressure and temperature to enhance the solubility and extraction of saponins in the solvent. The solvent is usually water or methanol. This process involves continuously passing solvent through the sample at high temperatures, thus avoiding boiling and allowing for optimal extraction in a short period of time [65].

**Figure 9:**
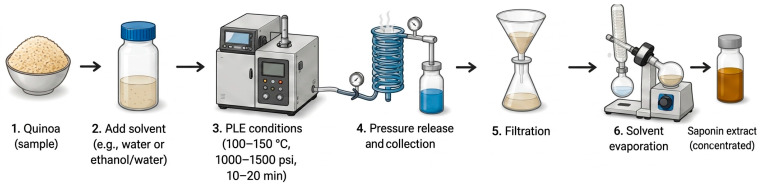
Infographic of pressurized liquid extraction. (Note: Figure generated using ChatGPT (OpenAI, GPT-5.5) and adapted by the authors).

High temperature and pressure keep the solvent in liquid form, enhancing the solubility and diffusion of saponins and reducing the extraction time (Figure 9).

**Figure 10:**
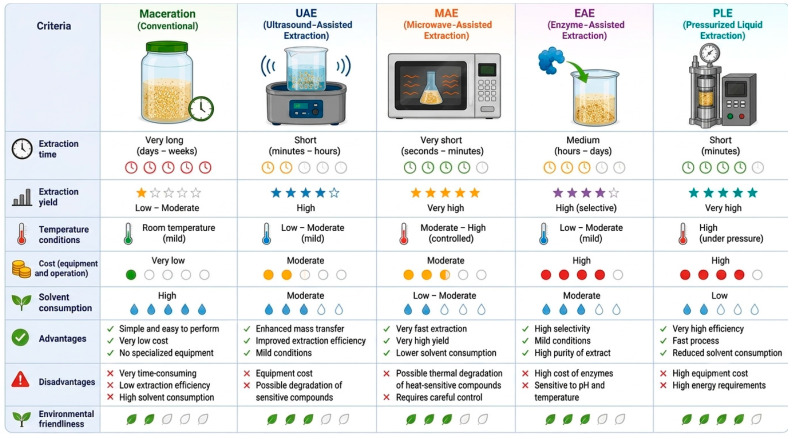
Comparison of extraction methods for quinoa saponins. (Note: Figure generated using ChatGPT (OpenAI, GPT-5.5) and adapted by the authors).

All extraction methods have advantages and disadvantages, as shown in Figure 10. The maceration method requires the longest extraction time, which can last from days to weeks, while the shortest time will be found in the MAE method, where we refer to seconds to receive the distillate. The extraction efficiency and the cost of each method are key selection criteria. Maceration is the least expensive method, but the extraction efficiency is very low compared to the other methods. The other methods range from high to very high extraction efficiencies, with EAE and PLE being the most expensive.

In conclusion, PLE is the most effective but with high cost and solvent consumption, while UAE and MAE offer fast and efficient extraction with moderate cost and energy consumption. UAE is suitable for sensitive materials. Ultrasound-assisted extraction and microwave-assisted extraction are considered the most suitable methods for quinoa saponins.

According to the data in Table 3, the MAE extraction method is the most efficient (22–32 mg/g dry weight) and least time-consuming (5–15 min), whereas maceration is the least efficient (10–25 mg/g dry weight) and most time-consuming (1–24 h). When considered alongside the data in Figure 10, MAE emerges as one of the costliest methods, like PLE, in contrast to UAE, which offers a moderate extraction time (20–90 min), good efficiency (20–30 mg/g dry weight), and lower cost.

## 5. Pharmacological Effects

### 5.1. Anti-Inflammatory Activity

Quinoa has been traditionally recognized for its medicinal properties. For the treatment of muscle strains, bruises, and joint injuries, black quinoa seeds have been used, which were mixed with alcohol and placed in the form of a poultice on the strained area, demonstrating their traditional anti-inflammatory applications [68]. Studies have suggested that quinoa saponins can suppress the production of inflammatory mediators, such as nitric oxide, indicating significant anti-inflammatory activity. The potential use of quinoa saponins as functional food ingredients with anti-inflammatory properties has been proposed [69]. Research results showed that saponins improved intestinal microflora dysbiosis in mice treated with them. Metabolic analysis identified UDCA (ursodeoxycholic acid) as a protective metabolite, which was subsequently found to target the TLR4/NF-κB signaling pathway, enhancing the antioxidant capacity of the colon and maintaining the integrity of the intestinal barrier [70].

### 5.2. Immunomodulatory Activity

Direct experimental data regarding the immunoadjuvant activity of quinoa saponins are currently limited. Consequently, the studies cited below concern saponins derived from plant species other than *Chenopodium quinoa* and are presented solely to provide general information on the immunoadjuvant properties and mechanisms of action of plant saponins. These findings should not be interpreted as direct evidence concerning quinoa saponins but rather as a broader context for future research.

Plant saponins have been investigated as immunological adjuvants in animal vaccine studies and have been reported to enhance both systemic and mucosal antibody responses following intragastric (IG) or intranasal (IN) administration [71]. It is widely accepted that oral or intranasal vaccination generally requires the repeated administration of relatively high antigen doses. However, it has been demonstrated that certain plant saponins enhance antibody responses at comparatively low doses and with antigens of minimal immunogenicity when administered via mucosal routes [72].

### 5.3. Anticancer Activity

Quinoa saponins have demonstrated potential anticancer effects on specific types of cancers such as of the colon and breast.

The number of patients with colon cancer has increased in recent years, growing steadily by about 3% per year. There are many research findings that have documented that the active ingredients of different plant species can inhibit the productivity of cancer cells’ proliferation and selectively target malignant tumors, which constitutes a new strategy for the treatment of tumors [73].

Studies examining quinoa saponins and their isolated sapogenins have indicated potential anticancer activity, primarily based on in vitro experimental models. The anticancer effects fall within the fact that saponins affect the distribution of the cell cycle, where they alter the expression of proteins in cells, thereby affecting the growth of cancer cells. Research has shown that cells treated with quinoa saponins were killed. In vitro study with quinoa saponins (40 μg/mL) significantly reduced the percentage of viable HT-29 cells and increased the apoptosis rate by 22.55% compared to the control group. In addition, the percentage of cells arrested in the G0/G1 phase increased by 22.97% [74,75]. Isolated quinoa saponins and sapogenins have been evaluated and found to exhibit anticancer activity. Specifically, derivatives of hederagenin, oleanolic acid, phytolaccagenic acid, and serjanic acid have been identified in the plant *Chenopodium quinoa* and studied for their cytotoxic activity [29,76].

Breast cancer remains the most commonly diagnosed cancer worldwide, accounting for approximately 11.7% of all new cancer cases. Previous studies have shown that quinoa saponins may induce apoptosis in cancer cell models. In addition, using saponin fractions from other plant species, such as *Quillaja saponaria* Molina, have demonstrated cytotoxic activity and the induction of apoptosis in cancer cell models [77]. The biological activity of saponins, including those derived from quinoa, is considered to be related to their amphiphilic structure. In particular, the lipophilic part of the triterpene aglycone favors the integration of saponins with cell membranes, while the hydrophilic sugar part allows them to bind to extracellular glycolipids and glycoproteins. Thus, they disrupt and destabilize the membrane [78].

## 6. Toxicity and Limitations

Quinoa saponins are often referred to as antinutritional factors due to their bitter taste and their potential to interfere with nutrient utilization at high concentrations. By this interpretation, we mean chemical compounds that reduce the nutritional value of a food, resulting in delayed or reduced digestion and non-absorption or utilization of nutrients [79].

*Chenopodium quinoa* saponins can induce hemolysis at high concentrations through membrane disruption, primarily via lipid solubilization or pore formation. However, hemolytic activity varies significantly among different saponins and depends on their chemical structure and concentration [80]. In vivo clinical studies have not recorded any adverse side effects at doses below 50 mg/kg body weight per day.

However, some types of saponins form compounds with iron or zinc, but there are no documented results proving that they can also form with vitamins. Current evidence indicates that there are research results supporting that quinoa saponins are antinutritional agents and exhibit toxic effects primarily at high concentrations [81].

Laboratory in vivo studies have shown that as triterpenoid saponins with amphiphilic activity, they have the ability to stimulate the digestive system at high administered doses, which are above 10 g per kg of body weight [79]. Saponins remain in the body for a long time, while their toxicity is limited. It is worth noting that the elimination of saponins from the seeds before consumption is mainly due to the bitter taste they impart rather than their potential toxicity.

As already mentioned, quinoa can be classified into sweet genotypes (without or with <0.11% saponin content, from 20 to 40 mg/100 g dry weight) or bitter genotypes (containing >0.11% saponin content, from 140 to 2300 mg/100 g dry weight) [81].

An upper limit for safe human consumption of saponins from quinoa seeds has been set, mostly due to the bitter taste they impart but also for certain antinutritional factors; this limit is <0.12% saponin content [82].

Compared to saponins from other plant species, quinoa saponins exhibit variable hemolytic activity, which depends largely on their chemical structure, the type of aglycone, the sugar moieties, and the concentration [83]. For example, *Quillaja saponaria*-derived saponins are known for their potent immunoadjuvant properties, but they may exhibit strong hemolytic activity at high concentrations [84].

## 7. Future Prospects

There are clinical studies with validated results on the therapeutic potential of quinoa saponins, and they have reported promising results. However, further research is needed to validate and improve their efficacy and safety in larger populations. Future clinical trials should focus on a broader range of diseases and conditions, particularly those associated with metabolic disorders, such as hypercholesterolemia, obesity, and cardiovascular disease. Future studies could be carried out regarding the mechanism of action, i.e., which specific triterpene saponin aglycones (e.g., oleanolic acid, ederagenin, and phytolaccagenic acid) are responsible for the observed anticancer or hypocholesterolemic effects? Structure–activity relationship (SAR) studies could be conducted using molecular binding and in vitro enzyme inhibition assays. In addition, dosage and bioavailability should be evaluated. What is the predicted equivalent dose for humans based on existing data from animal studies (e.g., no adverse effects below 50 mg/kg/day)? Co-administration with absorption enhancers is recommended, which should be tested to overcome the low bioavailability due to the polarity of saponins. Specifically, a 90-day repeated-dose oral toxicity study in two mammalian species (rodents and non-rodents), with histopathological examination of the intestinal mucosa, liver, and kidneys, is proposed, given the known hemolytic and membrane-damaging properties of saponins.

Finally, studies should contribute to a better public understanding of quinoa saponins. Future research should also investigate the development of pharmaceutical formulations and biomedical applications based on quinoa saponins. In particular, their potential use as vaccine adjuvants and anticancer formulations warrants further investigation. Although they are widely recognized as antinutritional compounds, increasing evidence suggests that they may exert beneficial biological activities when present in appropriate concentrations.

## 8. Conclusions

Quinoa (*Chenopodium quinoa* Willd.) saponins constitute an important group of bioactive triterpenoid compounds with significant medicinal and nutritional potential. Although traditionally considered antinutritional agents due to their bitter taste and potential toxicity at high concentrations, increasing scientific evidence has demonstrated a wide range of beneficial biological activities, including antioxidant, anti-inflammatory, antimicrobial, and immunomodulatory effects. Advances in extraction technologies have also improved the recovery and utilization of these compounds for potential industrial and biomedical applications. Despite these promising findings, most of the evidence so far comes from in vitro and animal studies.

Overall, the findings examined in this review support the premise that quinoa saponins possess valuable pharmacological properties beyond their traditional classification as antinutritional compounds. However, further preclinical and clinical studies are required to confirm their efficacy, safety, and mechanisms of action before their potential therapeutic applications can be realized.

## Figures and Tables

**Table 1:** Macronutrients contained in quinoa seeds.

Component	Average Range per 100 g (Dry Weight)	Nutritional Significance	Reference
Carbohydrates	(53.2–73.4%)	Mainly low-glycemic starch (58–64%).	[38]
Proteins	(11.3–18.9%)	Rich in albumin and globulin; includes lysine.	[38]
Dietary Fiber	(7.7–18.59%)	Comparable to legumes; aids digestion.	[38]
Crude Fat (Lipids)	(4.0–7.09%)	Mostly healthy unsaturated fatty acids.	[38]
Moisture	(9.68–12.62%)	Standard raw seed shelf-stable humidity.	[39]
Ash (Total Minerals)	(2.79–5.46%)	High presence of inorganic mineral residues.	[39]

**Table 2:** Micronutrients contained in quinoa seeds [40].

Mineral	Average Range per 100 g (Dry Weight)	Functional Role
Potassium (K)	Up to (1040.0 mg)	Critical fluid balance and heart health regulator.
Phosphorus (P)	(406.0 mg–408.3 mg)	Crucial component for cellular energy and bone density.
Magnesium (Mg)	(147.0 mg–204.2 mg)	Supports healthy muscle and metabolic nervous systems.
Calcium (Ca)	(23.0 mg–66.6 mg)	Essential for structural bone maintenance.
Iron (Fe)	(2.6 mg–10.9 mg)	Essential micro-mineral that helps prevent anemia.
Zinc (Zn)	Up to (7.47 mg)	Key structural element for functional immune defenses.

**Table 3:** Representative bibliographic values of extraction time and total saponin yield for different extraction methods from quinoa seeds [66,67].

Extraction Method	Typical Extraction Time	Reported Total Saponin Yield (mg/g Dry Weight)
Maceration	1–24 h	10–25
Ultrasound-assisted extraction (UAE)	20–90 min	20–30
Microwave-assisted extraction (MAE)	5–15 min	22–32
Pressurized liquid extraction (PLE)	15–30 min	22–35
Enzyme-assisted extraction (EAE)	1–3 h	18–30

## Data Availability

No new data were created or analyzed in this study. Data sharing is not applicable to this article.

## Abbreviations

The following abbreviations are used in this manuscript:

EAE	Enzyme-Assisted Extraction
FAO	Food and Agriculture Organization of the United Nations
G0/G1	Gap 0/Gap 1 Phase of the Cell Cycle
HT-29	Human Colorectal Adenocarcinoma Cell Line
IG	Intragastric
IN	Intranasal
MAE	Microwave-Assisted Extraction
NF-κB	Nuclear Factor Kappa B
PLE	Pressurized Liquid Extraction
SAR	Structure–Activity Relationship
TLR4	Toll-Like Receptor 4
UAE	Ultrasound-Assisted Extraction
UDCA	Ursodeoxycholic Acid
